# Mechanism and Characterization of Bicomponent-Filler-Reinforced Natural Rubber Latex Composites: Experiment and Molecular Dynamics (MD)

**DOI:** 10.3390/molecules30020349

**Published:** 2025-01-16

**Authors:** Zhipeng Feng, Hongzhou Zhu, Bo Hu, Huabin Chen, Yong Yan

**Affiliations:** 1 Broadvision Engineering Consultants Co., Ltd., Kunming 650200, China; jabinfeng@gmail.com (Z.F.);; 2 School of Civil Engineering, Chongqing Jiaotong University, Chongqing 400074, China; 3 National & Local Joint Engineering Research Center of Transportation and Civil Engineering Materials, Chongqing Jiaotong University, Chongqing 400074, China; 4 Faculty of Civil Engineering and Mechanics, Kunming University of Science and Technology, Kunming 650032, China; 5 Yunnan Key Laboratory of Disaster Reduction in Civil Engineering, Kunming 650500, China

**Keywords:** natural rubber latex, multi-walled carbon nanotubes, silica, dispersion behavior, mechanical properties, molecular dynamics simulation

## Abstract

The incorporation of reinforcing fillers into natural rubber latex (NR) to achieve superior elasticity and mechanical properties has been widely applied across various fields. However, the tendency of reinforcing fillers to agglomerate within NR limits their potential applications. In this study, multi-walled carbon nanotube (MWCNT)–silica (SiO_2_)/NR composites were prepared using a solution blending method, aiming to enhance the performance of NR composites through the synergistic effects of dual-component fillers. The mechanical properties, dispersion behavior, and Payne effect of three types of composites—SiO_2_/NR (SNR), MWCNT/NR (MNR), and MWCNT-SiO_2_/NR (MSNR)—were investigated. In addition, the mean square displacement (MSD), fractional free volume (FFV), and binding energy of the three composites were simulated using molecular dynamics (MD) models. The results showed that the addition of a two-component filler increased the tensile strength, elongation at break, and Young’s modulus of NR composites by 56.4%, 72.41%, and 34.44%, respectively. The Payne effect of MSNR was reduced by 4.5% compared to MNR and SNR. In addition, the MD simulation results showed that the MSD and FFV of MSNR were reduced by 21% and 17.44%, respectively, and the binding energy was increased by 69 times, which was in agreement with the experimental results. The underlying mechanisms between the dual-component fillers were elucidated through dynamic mechanical analysis (DMA), a rubber process analyzer (RPA), and field emission scanning electron microscopy (SEM). This study provides an effective reference for broadening the application fields of NR.

## 1. Introduction

Natural rubber latex plays an important role in the global economy, with more than 13 million tons of natural rubber latex produced globally each year to meet the needs of a wide range of industries, particularly the transportation, medical, and manufacturing sectors. Natural rubber latex (NR) is widely used in tires, industrial products, and essential daily products due to its excellent comprehensive properties [1,2,3,4,5]. However, the inherent properties of pure NR are limited, restricting its broader application. Fillers, as an important component of rubber composites, play a critical role in enhancing the performance of rubber composites. Reinforcing fillers such as carbon black (CB) [6,7,8,9], carbon nanotubes (CNTs) [10,11], and silica (SiO_2_) [12,13,14] are commonly used in NR. However, integrating separate reinforcing fillers into natural rubber latex, while significantly enhancing certain properties, is a high-cost process that suffers from processing difficulties and leads to poor dispersion and increased brittleness [15], seriously affecting the practical application of natural rubber latex composites. Thus, there is a need to improve the dispersion of reinforcing fillers in matrices to expand the application scope of natural rubber latex to applications such as high-performance materials, conductive rubber, electronic packaging, and high-temperature and aging applications.

To address the above-mentioned issues, researchers have focused on modifying reinforcing fillers to enhance their dispersion in the matrix. For instance, You et al. [16] used a silane coupling agent (Si-69) to perform a hydrophobic modification of SiO_2_. The organic groups of Si-69 improved the interfacial compatibility between SiO_2_ and rubber, increasing the affinity between the two and reducing the aggregation and agglomeration of SiO_2_ particles. This improved its dispersion within NR and enhanced the mechanical properties of NR. Zhou et al. [17] utilized ionic liquids (ILs) and 1-ethyl-3-methylimidazole acetate to introduce carbon black (CB) homogeneously into NR to optimize its structure and properties, resulting in a reduced mutual attraction between particles, an increased interfacial compatibility, and improved rheological properties. Xun et al. [18] oxidized CNTs using potassium ferrate (K_2_FeO_4_), increasing the number of surface hydroxyl groups, which allowed for CNTs to be uniformly dispersed in NR and, thereby, enhanced the performance of NR composites. However, the above-mentioned modification processes are complex, are inefficient, and involve the use of large amounts of chemical reagents, which contribute to environmental pollution.

The method of using dual-component fillers to enhance polymer composites has been widely applied in various studies. For instance, Mohd Khairuddin et al. [19] used a combination of SiO_2_ and calcium carbonate (CaCO_3_) to generate micro-roughness, thereby improving the dispersion of SiO_2_ within the NR matrix and enhancing the hydrophobicity of NR composites. Shahamatifard et al. [20] combined carbon black (CB) with multi-walled carbon nanotubes (MWCNTs) to study the dispersion, mechanical properties, and thermal conductivity of CB and MWCNTs within NR, showing that both the thermal conductivity and mechanical properties of the composites were improved. Sun et al. [21] synthesized low-cost crude carbon dots (CDDs) mixed with SiO_2_ via an industry-compatible melt blending method to improve the vulcanization rate and mechanical properties of NR. However, these studies all require specialized equipment, and the preparation processes are complex, making practical engineering applications challenging. Meanwhile, there is a lack of relevant computational simulations in the above studies—they mainly focus on experimental work. Therefore, it is of great significance to design NR composites with a simple preparation process and good dispersion using a combination of experiments and computational simulations.

Molecular dynamics (MD) simulations have become an effective computational tool for studying the physical behavior and molecular interactions of materials at the atomic scale. They can complement experimental research and provide a strong theoretical basis for studies. MD simulations have proven to be suitable for investigating polymer material properties, including their binding energies and intermolecular interaction forces [22,23,24,25,26,27]. The application of molecular dynamics simulations in filler dispersion studies is very important because they provide a deep understanding of the interaction between the filler and the matrix and reveal the dynamic processes of distribution, aggregation, and movement of the filler at the molecular level. Therefore, a comprehensive study combining experimental methods and MD simulations is necessary to evaluate the applicability of NR enhanced with dual-component fillers.

MWCNTs have a very high surface area and excellent electrical conductivity, leading to excellent interfacial bonding with SiO_2_. Due to the strong van der Waals forces and π-π interactions, carbon nanotubes can effectively promote the dispersion of the fillers and form strong and effective contacts with the SiO_2_ surface, which enhances the stability and homogeneous distribution of the fillers in the matrix. Compared with single fillers or other traditional two-component fillers (e.g., the combination of carbon black and SiO_2_), the composite system of MWCNTs and SiO_2_ is capable of achieving a more homogeneous dispersion and reducing aggregation phenomena at the microscopic scale. In this study, SiO_2_ and MWCNTs were used as reinforcing fillers, with NR as the matrix. The aim was to enhance the interfacial bonding between the NR matrix and the fillers by employing dual-component fillers to reinforce the NR composites and to improve the dispersion of these reinforcing fillers. NR composites with excellent mechanical properties and dispersion abilities were prepared using a solution blending method. The dispersion of the dual-component fillers within the NR was analyzed through dynamic mechanical analysis (DMA), rubber process analysis (RPA), and field emission scanning electron microscopy (SEM). Moreover, MD simulations further verified the consistency between the experimental results and molecular dynamics modeling. This research enables the combination of MWCNTs and SiO_2_ with potential for applications in high-performance tires, electronics, and high-temperature and chemical-resistant sealants.

## 2. Results

### 2.1. Mechanical Properties

**Figure 1:**
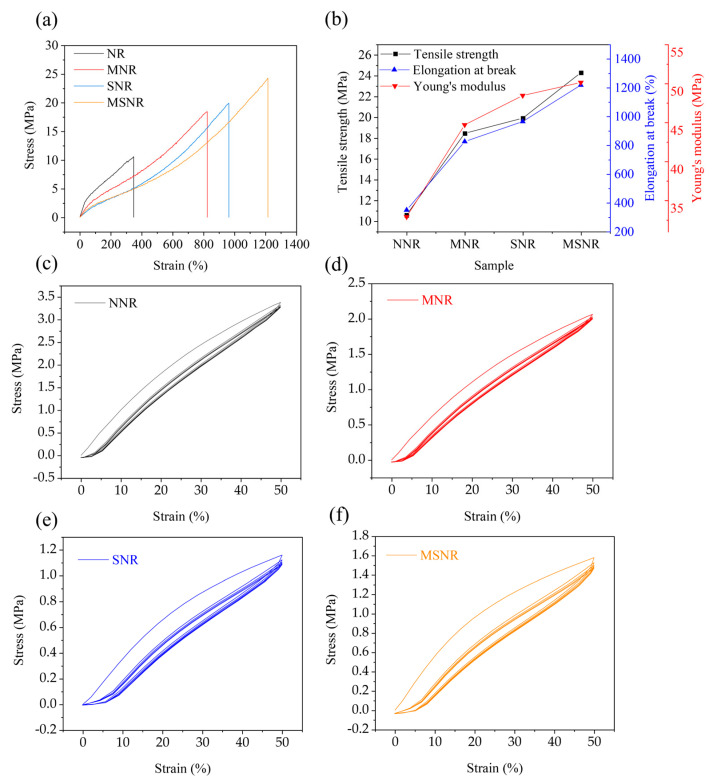
Mechanical properties of NNR, MNR, SNR, and MSNR: (**a**) stress–strain curve, (**b**) tensile strength, (**c**–**f**) stress–strain hysteresis curves.

Good mechanical properties are crucial for practical applications. Figure 1a shows the stress–strain curves of the four composites, all of which exhibit a linear growth trend. Among them, MSNR shows the most pronounced growth curve, indicating increased stiffness when dual-component fillers are added. Figure 1b presents the tensile strength, elongation at break, and Young’s modulus of the four composites. It is evident that MSNR has the highest tensile strength, which is attributed to the synergistic effect between the MWCNTs and SiO_2_ that reduces filler agglomeration in the NR matrix, allowing for the fillers to be better dispersed. Upon stretching, dispersing stress is generated, resulting in MSNR having an excellent tensile strength. In addition, MSNR exhibits the highest elongation at break, indicating that this material can withstand higher stress concentrations without failure. Furthermore, the Young’s modulus of MSNR was increased by 28.74%, 3.35%, and 3.54% compared to NNR, SNR, and MNR, respectively, demonstrating that MSNR has a good resistance to deformation. Specific mechanical properties are shown in Table S2, and compared with other studies on the mechanical properties of natural rubber latex, the reinforcement effect of the two-component filler is better [2,5,7,16,17,18].

Figure 1c–f show the cyclic stress–strain curves of NNR, SNR, MNR, and MSNR. It can be observed that for all four composites, the area enclosed by the curves decreases with an increasing number of cycles, eventually converging. This is due to the viscoelastic nature of rubber materials, where deformation increases with strain, and upon unloading, the rubber cannot fully return to its original state, resulting in residual deformation [28].

Stress–strain cycling curves were used to evaluate energy loss in composites. A larger hysteresis area indicates a higher energy loss in the material, which is due to the friction generated between the filler and the NR molecular chains when strain occurs [29]. The initial stress–strain cycling curves of the extracted NNR, SNR, MNR, and MSNR are shown in Figure S1a–d. It can be seen that the hysteresis area of MSNR is the largest, and the energy loss within MSNR is large enough to effectively absorb and consume the externally applied energy, thus effectively reducing the propagation of vibrations and impact energy.

### 2.2. Rheological Properties

**Figure 2:**
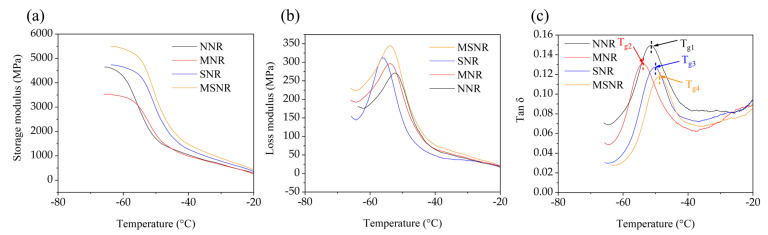
Rheological properties of NNR, MNR, SNR, and MSNR: (**a**) storage modulus, (**b**) loss modulus, (**c**) loss factor.

DMA provides insight into the mechanical response of rubber materials under various operating conditions, especially under dynamic loading. Rubber composites are commonly used in applications such as automotive tires, shock absorbers, and seals, where the materials are subjected to complex loading conditions. DMA is an important tool for studying the properties of materials under these complex conditions. Figure 2a shows the storage modulus of NNR, SNR, MNR, and MSNR at different temperatures. It is evident that the storage modulus of MSNR is higher than that of NNR, SNR, and MNR, indicating that the dual-component fillers improved the dispersion of the fillers within the NR matrix, enhancing the internal cohesion and interfacial bonding capabilities of NR. This contributes to extending the service life of NR and reducing deformation-related damage during use. Figure 2b presents the loss modulus of the four composites. Compared to NNR, SNR, and MNR, MSNR exhibits a higher loss modulus, indicating better filler dispersion and enhanced interfacial interactions within the NR matrix. Figure 2c illustrates the loss factor of the four composites, with MSNR showing the lowest loss factor, suggesting that the dual-component fillers enable efficient energy storage and recovery within the NR matrix, resulting in better elastic behavior under dynamic deformation. This also indicates that it maintains good elasticity at low temperatures, thus preventing material embrittlement and making it suitable for low-temperature environments. MSNR has good energy absorption and vibration damping properties, demonstrating that it has a good resistance to deformation. Specific DMA properties are shown in Table S3. Tg1, Tg2, Tg3, and Tg4 represent the glass transition temperatures of NNR, MNR, SNR, and MSNR, respectively. It is evident that Tg4 shifts significantly to the right, which is due to the introduction of dual-component fillers that reduce the mobility of NR, restrict the free movement of NR chains, and lead to stronger interfacial bonding [30,31]. Specific rheological property ratios are shown in Table S2.

**Figure 3:**
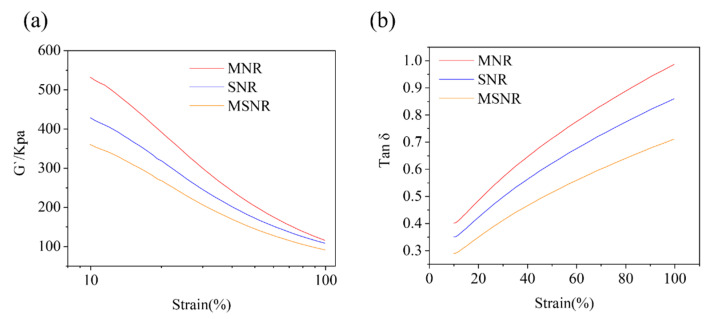
Rheological properties of MNR, SNR, and MSNR at 100% strain: (**a**) storage modulus, (**b**) loss modulus.

The dispersion performance of MNR and MSNR was evaluated using a rubber process analyzer (RPA). Figure 3a shows the storage modulus of the three composites under strain. It is evident that all three composites exhibit a decreasing curve as strain increases, which is due to the destruction of the internal crosslinked network structure of the composites—a phenomenon known as the Payne effect [32]. The Payne effect is a significant non-linear change in the modulus of elasticity of a rubber or other polymer-based composite materials when a load is applied. This phenomenon is particularly evident in rubber matrix composites and is often used to describe the effect of the dispersion state of the filler on the properties of the material. In the case of tires, the Payne effect has an important influence on the nonlinear properties exhibited by the material under load. The nonlinear stiffness of the material can affect the traction, fuel efficiency, and wear behavior of the tire. A lower Payne effect indicates that the filler is more uniformly dispersed in the rubber matrix, forming fewer aggregates or agglomerated structures. This is due to the lower interaction force between filler particles, which avoids the localized increase in stiffness due to aggregation, thus reducing the dynamic modulus of the composite. The value of ΔG’ = G’ (10.47%) − G’ (95.35%) was used as a standard to evaluate dispersion, where ΔG’ represents the difference in G’ between a 10.47% strain and 95.35% strain. A larger ΔG’ value indicates more severe filler agglomeration in the matrix [32]. The calculated ΔG’ values for MNR, SNR, and MSNR are 417.27, 320.87, and 269.56, respectively, indicating that the dual-component fillers in MSNR are more uniformly dispersed in the NR matrix. Furthermore, Figure 3b shows the loss factor of MNR, SNR, and MSNR under strain. It can be observed that the loss factor of MSNR is lower than that of MNR and SNR, which is due to the complementary effect of the dual-component fillers within the matrix during deformation, improving the dispersion of MWCNTs in NR and optimizing the crosslinked network of NR.

### 2.3. Analysis of Interfacial Interaction Using the Lorenz–Park Method

**Figure 4:**
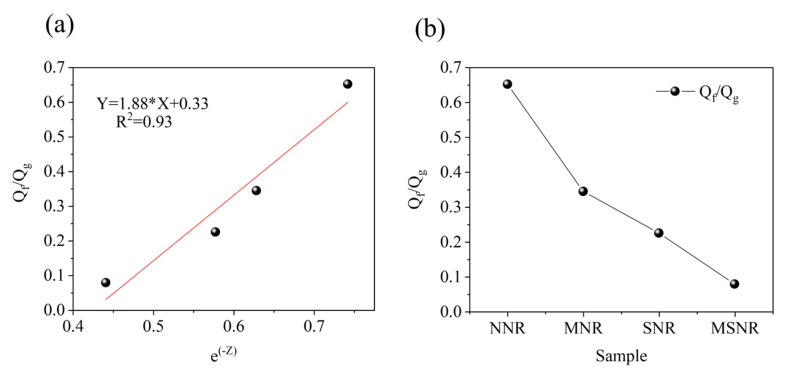
(**a**) Trend plot of *Q_f_*/*Q_g_* as a function of *e*^−*Z*^ for four composites. (**b**) Trend plot of *Q_f_*/*Q_g_* for four composites.

The interfacial interactions between the filler and matrix were evaluated using the equations proposed by Lorenz and Park. Figure 4a shows the plot of *Q_f_*/*Q_g_* as a function of *e*^−*Z*^ using the NNR, MNR, SNR, and MSNR composite specimens. The values of parameters a and b are constants of the equation with values of 1.88 and 0.33, respectively, giving a correlation coefficient (R) of 0.92. According to the observation of Lorentz and Park, a constant value higher than 0.7 indicates a strong interaction between the MWCNTs and SiO_2_ filler and the NR matrix [33]. In Figure 4b, the decrease in *Q_f_*/*Q_g_* values with the introduction of MWCNTs and SiO_2_ is evident, confirming the effective interaction between the rubber matrix and filler.

### 2.4. Microscopic Morphology

**Figure 5:**
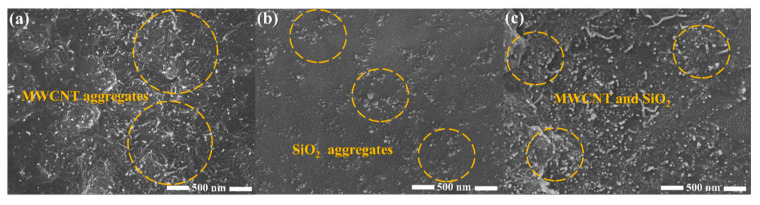
Microscopic morphology by SEM. (**a**) MNR, (**b**) SNR, and (**c**) MSNR.

The dispersion of fillers within the NR matrix is crucial. Figure 5a shows the SEM image of MNR, where it is evident that MWCNTs are entangled within the NR matrix, hindering the uniform dispersion of the filler within the matrix and leading to severe agglomeration. Figure 5b shows the SEM image of SNR. Compared to MNR, the SiO_2_ in SNR is more uniformly dispersed, though some minor agglomerates are still present. In contrast, Figure 5c shows the SEM image of MSNR, where uniform dispersion within the NR matrix is observed. This is attributed to the high adsorption energy of the SiO_2_ surface, which leads to the attachment of MWCNTs to SiO_2_ and results in a synergistic effect that enhances the overall performance of NR. This synergy is also a key factor contributing to the excellent mechanical properties of MSNR.

### 2.5. Molecular Dynamics

#### 2.5.1. Mean Square Displacement (MSD, Section S2.1)

**Figure 6:**
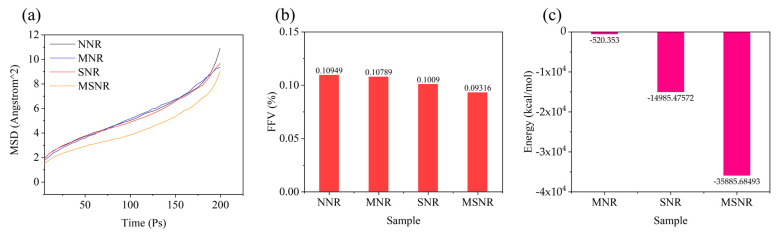
(**a**) MSD of NNR, MNR, SNR, and MSNR. (**b**) FFV of NNR, MNR, SNR, and MSNR. (**c**) Energy of MNR, SNR, and MSNR.

The mean square displacement (MSD) of polymer composites is used to represent the trends in the molecular chain motion in a system, defined as the distance between the positions of particles at time *t* and their initial positions [34]. It is typically calculated using the formula:

(1) $$\mathrm{MSD}=< {\left|r\left(t\right)-r\left(0\right)\right|}^{2} >$$

where $r\left(0\right)$ and $r\left(t\right)$ represent the position vectors of atoms at the initial time and time *t*, respectively, and <> denotes the average overall atoms in the system. Figure 6a shows the MSD of the four composites, indicating that MSNR has the lowest MSD, which suggests that the dual-component fillers aid in dispersion within the NR matrix and improve the strength of NR composites. Moreover, a lower MSD indicates a more restricted molecular chain movement, demonstrating that the dual-component fillers exhibit better interfacial bonding capabilities with the NR matrix [35].

#### 2.5.2. Fractional Free Volume (FFV, Section S2.2)

Figure 6b shows the fractional free volume (FFV) of the four composites. This parameter reflects the size and mobility of the free space available for molecular chains within polymer composites, which subsequently affects material performance [36]. The *FFV* is calculated using the following formula:

(2) $$FFV=\frac{V_{Free}}{V_{Total}}\times 100\%$$

where $V_{Free}$ and $V_{Total}$ are the free and total volumes of the model system, respectively. The *FFV* calculation models for the four composites are shown in Figure S2. The calculated *FFV* values exhibit a decreasing trend, with MSNR having the lowest *FFV*, indicating that the dual-component fillers reduce the free movement space of NR molecular chains, strengthen intermolecular interactions between the fillers, and more effectively restrict the movement of NR chains. This leads to NR composites having improved interfacial bonding and an enhanced strength.

#### 2.5.3. Binding Energy (Section S2.3)

The binding energy is used to characterize the strength of interactions between substances. It is calculated using the formula:

(3) $$E_{int}=E_{A+B}-\left(E_{A}+E_{B}\right)$$

where $E_{int}$ is the total interaction energy and $E_{A}$ and $E_{B}$ are the energies of individual components. When $E_{int}<0$, the interaction between materials is stronger [37]. Figure 6c shows the binding energy of MNR, SNR, and MSNR composites. The results indicate that MSNR exhibits the highest negative binding energy, suggesting improved interactions between the fillers and the polymer, leading to better dispersion within the NR matrix [37].

The results of the experimental data show that MSNR has significant advantages over other composites in terms of mechanical properties, energy storage modulus, loss modulus, and dispersion of filler in a rubber matrix. Furthermore, by comparing the molecular dynamics simulations, the volume fraction (FFV) and mean square displacement (MSD) of MSNR reached the minimum values, and the binding energy showed the maximum; these results demonstrate the strong interaction and high binding efficiency of MWCNT and SiO_2_ with the rubber matrix at the microscopic scale. This agreement between experimental and simulation results not only proves the reliability of the simulation method, but also highlights the remarkable potential of MWCNT and SiO_2_ bicomponent fillers to enhance the properties of natural rubber, which provides an important theoretical and experimental basis for the design and development of high-performance rubber matrix composites.

## 3. Materials and Methods

### 3.1. Materials

Polymers: natural rubber latex (NRL, with a solid content of 60%) was purchased from Zhengmao Petrochemical Co., Ltd., Maoming, Guangdong, China. Fillers: multi-walled carbon nanotubes (MWCNTs, length: 10–20 mm, diameter: 4–6 nm, specific surface area: 500–700 m^2^/g, purity > 98%) were obtained from Chengdu Organic Chemicals Co., Ltd., Chengdu, China. Silica (SiO_2_, particle size: 50 ± 5 nm) was purchased from Shanghai Macklin Biochemical Co., Ltd., Shanghai, China. Additives: other reagents, including sulfur powder (S), zinc oxide (ZnO), stearic acid (SA), *N*-isopropyl-*N*′-phenyl-*p*-phenylenediamine (4010NA), *N*-tert-butyl-2-benzothiazolesulfenamide (NS), and tetrahydrofuran (THF), were obtained from Kunming Kerei Instruments Co., Ltd., Kunming, China.

### 3.2. Sample Preparation

**Figure 7:**
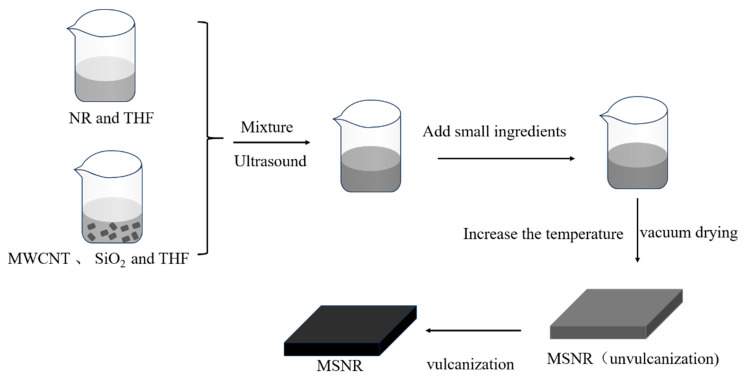
Flow chart of MSNR preparation.

In this study, NR composites were prepared using a solution blending method. First, NR, MWCNTs, and SiO_2_ were separately ultrasonically stirred in THF for 2 h; the rotational speed of the mixer and the ultrasonic power of the ultrasonic cleaner were 400 Rpm and 100 Hz, respectively. Subsequently, the materials required for vulcanization (S, ZnO, SA, NS, 4010NA) were added, and the mixture was heated to 50 °C to remove most of the solvent and vacuum-dried in an oven at 60 °C to remove all of the solvent to obtain an uncured MWCNT-SiO_2_/NR composite. Finally, the uncured MWCNT-SiO_2_/NR composites were vulcanized at a temperature of 150 °C and a pressure of 15 MPa for 15 min. The experimental formulation consisted of 4 g of NR, 0.08 g MWCNT, 0.2 g SiO_2_, 0.12 g sulfur (S), 0.2 g zinc oxide (ZnO), 0.08 g stearic acid (SA), 0.08 g *N*-isopropyl-*N*′-phenyl-p-phenylenediamine (4010NA), and 0.06 g *N*-tert-butyl-2-benzothiazolesulfenamide (NS). Pure NR was designated as NNR, SiO_2_/NR composites as SNR, MWCNT/NR composites as MNR, and MWCNT-SiO_2_/NR composites as MSNR. The experimental procedure is illustrated in detail in Figure 7. Experiment-specific formulations are presented in Table S4.

### 3.3. Characterization Method

#### 3.3.1. Mechanical Property Tests

The mechanical properties of the four samples were tested using a universal testing machine (WDW-10, Changchun Institute of Testing Machines, Changchun, China); knowing these properties is essential for materials used in practical applications. The experimental conditions were as follows: room temperature, rate: 200 mm/min^−1^, size: 40 × 10 × 1 mm. The motion of the molecular chains of the composites was investigated using a dynamic mechanical analyzer (DMA, TAQ800, TA Instruments, New Castle, DE, USA) to characterize the confined regions formed within the composites. The test temperature range and frequency were −140–50 °C and 1 Hz, respectively.

#### 3.3.2. Dispersion Performance Test

The Payne effect of the composites and the dispersion of fillers in the NR matrix were studied using a rubber processing analyzer (RPA, Premier RPA, Premier Control Technologies, Manchester, UK) with a strain range of 10–100% and a frequency of 1 Hz. A field emission scanning electron microscope (FE-SEM, NOVA Nano SEM450, Hillsboro, OR, USA) was used to characterize the morphology of the MWCNT/NR composites under an accelerating voltage of 10 kV. The samples were frozen in liquid nitrogen for 5 min, and then an external force was applied to fracture the samples to obtain a new fracture surface. The fracture specimen was sprayed with platinum to increase its conductivity and obtain higher-quality FE-SEM images.

#### 3.3.3. Analysis of the Interfacial Interaction Between the MWCNTs and the Matrix (Via the Lorenz–Park Method)

Analysis of the Interfacial Interaction between the MWCNTs, SiO_2_, and the NR Matrix (via the Lorenz–Park Method).

The Lorenz–Park method was used to evaluate the interaction between MWCNTs, SiO_2_, and NR [38]. Based on swelling tests conducted in a solvent, the appropriate parameters were determined, and the interaction was calculated using Equation (2) [33]:

(4) $$\begin{array}{c} \;\frac{Q_{f}}{Q_{g}}=ae^{-z}+b \end{array}$$

Assume *Q* is the mass of toluene absorbed per gram of rubber; f and g represent the vulcanized nanocomposite including a filler and NR, respectively; and *z* represents the ratio of the filler mass to the rubber mass. *a* and *b* are constants. Equation (3) is used for the calculation.

(5)  Q=ωs−ωdωr × 100ωF

where $\omega_{s}$ refers to the mass of the swollen composite when equilibrium is reached, $\omega_{d}$ refers to the mass of the dry composite, $\omega_{r}$ refers to the mass of the rubber in the dry composite, and $\omega_{f}$ refers to the total mass of the formulation.

### 3.4. Molecular Dynamics Simulation Methods

**Figure 8:**
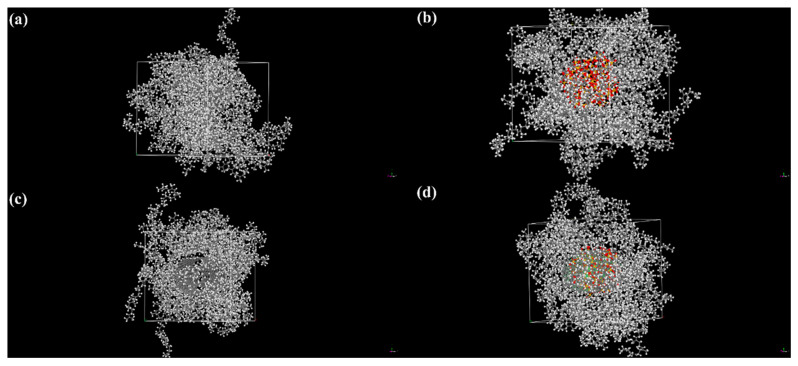
Image of molecular dynamics simulation. (**a**) NNR, (**b**) SNR, (**c**) MNR, and (**d**) MSNR.

In this study, Materials Studio 2020 (MS) software was used for molecular dynamics (MD) simulations to construct polymer models and periodic structures. The main component of natural rubber latex is cis-1,4-polyisoprene, and repeat units were created using the Visualizer module in MS, with a polymerization degree of 20 [39,40] to establish MWCNTs and SiO_2_. MWCNT parameters were set at N: 6 and M: 6, with 10 repeat units, where N and M are the diameters of the outer and inner carbon nanotubes, respectively. The radius of SiO_2_ was set to 10 Å, as shown in Figure S3a–d. A 40 × 40 × 40 empty cubic unit cell was constructed, and the Amorphous Cell module was used to build natural rubber latex molecular chains, with a predefined density of 0.93 m^2^/g, through the Construction function. A single-repeating-unit molecular chain was placed into the unit cell. The Packing module was then used to separately incorporate MWCNTs and SiO_2_ into the unit cell; the models for NNR, SNR, MNR, and MSNR are shown in Figure 8a–d. Subsequently, the lowest energy model was obtained using the Forcite module for geometry optimization and annealing (300–500 K, heating ramps per cycle: 5, dynamics steps per ramp: 100, total number of steps: 50,000) and dynamics simulations (Time step: 1 fs, Total simulation time: 200 ps, Number of steps: 20,000). Finally, the fractional free volume (FFV), mean square displacement (MSD), and binding energy of the four models were calculated. The COMPASS force field was used throughout the above process, with a time step of 1 fs and a total simulation time of 200 fs, under an NVE ensemble.

## 4. Conclusions

The objective of this study was to improve the dispersion of fillers within a natural rubber latex (NR) matrix. NR composites, multi-walled carbon nanotube (MWCNT)/NR (MNR) composites, silica (SiO_2_)/NR (SNR) composites, and MWCNT-SiO_2_/NR (MSNR) composites were prepared using a solution blending method to investigate the effects of dual-component MWCNT and SiO_2_ fillers on NR composites. Mixing multi-walled carbon nanotubes with silica in a natural rubber latex matrix produces a significant synergistic effect and improves the mechanical and dispersive properties, making this composite material suitable for a wide range of applications in the fields of automobile tires, conveyor belts, and electrical insulation materials. The following conclusions were drawn:

(1) After adding MWCNTs and SiO_2_, the tensile strength, elongation at break, and Young’s modulus of the NR composites increased by 59.85%, 71.44%, and 28.74%, respectively.

(2) Dynamic mechanical analysis (DMA) showed that the storage modulus and loss modulus of NR composites increased after adding MWCNTs and SiO_2_, while the loss factor decreased. This indicates that the stiffness of MSNR increased, and the molecular chains within NR were restricted, resulting in a higher interfacial bonding strength.

(3) The dispersion performance of the MNR, SNR, and MSNR composites was analyzed using a rubber process analyzer (RPA). The Payne effect of the MSNR composite was lower, indicating better dispersion.

(4) The fracture surface morphology of the MNR, SNR, and MSNR composites was analyzed using scanning electron microscopy (SEM). The synergistic effect between MWCNTs and SiO_2_ in the MSNR composite resulted in a uniform distribution of all fillers within the NR matrix, thereby improving the overall performance of NR.

(5) Molecular dynamics (MD) simulations were used to calculate the MSD, FFV, and binding energy of the composites. The results showed that MSNR had the lowest MSD and FFV and the highest negative binding energy, indicating that the dual-component fillers restricted the movement of NR molecular chains and exhibited better interfacial bonding with the NR matrix. These simulation results were consistent with the experimental findings.

(6) The hysteresis area of the first cycle stress–strain curve of MSNR was the largest; a larger hysteresis area usually means that the material loses more energy after a force is applied, resulting in a larger hysteresis loss. The results show that MSNR has a weak elastic recovery compared to the other three composites. This is due to the fact that NR is inherently viscoelastic. In future work, we will focus on the difficult task of reducing the viscoelasticity of NR composites to minimize the hysteresis loss.

## Data Availability

Data can be made available by the corresponding author upon request.

## Supplementary Materials

The following supporting information can be downloaded at: https://www.mdpi.com/article/10.3390/molecules30020349/s1, Figure S1: Stress–strain cyclic curve of NNR, MNR, SNR and MSNR; Figure S2: Free volume model of NNR, MNR, SNR and MSNR. Figure S3: Molecular dynamics model: (a) NR; (b) MWNCT; (c) SiO_2_ and (d) different atoms. Table S1: Definitions of abbreviation used in this study; Table S2: Comparison of mechanical properties. Table S3: DMA performance comparison. Table S4: The formula of the composites.
