# Supplementary material for: Mechanism and Characterization of Bicomponent-Filler-Reinforced Natural Rubber Latex Composites: Experiment and Molecular Dynamics (MD)
Source: Molecules. 2025 Jan 16;30(2):349. doi: 10.3390/molecules30020349 (PMC11767476; doi:10.3390/molecules30020349)
Supplement: Supplementary file 1 [file molecules-30-00349-s001.zip › molecules-3397765-supplementary.pdf]

Supplementary Materials

Table S1. Definitions of abbreviation used in this study.

Full title	Abbreviation
Natural rubber latex latex	NRL
Multi-walled carbon nanotube	MWCNT
Silica	SiO_2
Sulfur powder	S
Zinc oxide	ZnO
Stearic acid	SA
N-isopropyl-N′-phenyl-p-phenylenediamine	4010NA
N-tert-butyl-2-benzothiazolesulfenamide	NS
Tetrahydrofuran	THF
Carbon black	CB
Silane coupling agent	Si-69
Potassium ferrate	K_2FeO_4
Ionic liquid	IL
Crude carbon dots	CDDs
Calcium carbonate	$CaCO_3$
Dynamic mechanical analysis	DMA
Rubber process analysis	RPA
Scanning electron microscopy	SEM
Molecular dynamics simulations	MD
Mean square displacement	MSD
Fractional free volume	FFV
SiO_2/NR	SNR
MWCNT/NR	MNR
MWCNT-SiO_2/NR	MSNR

S2 Results

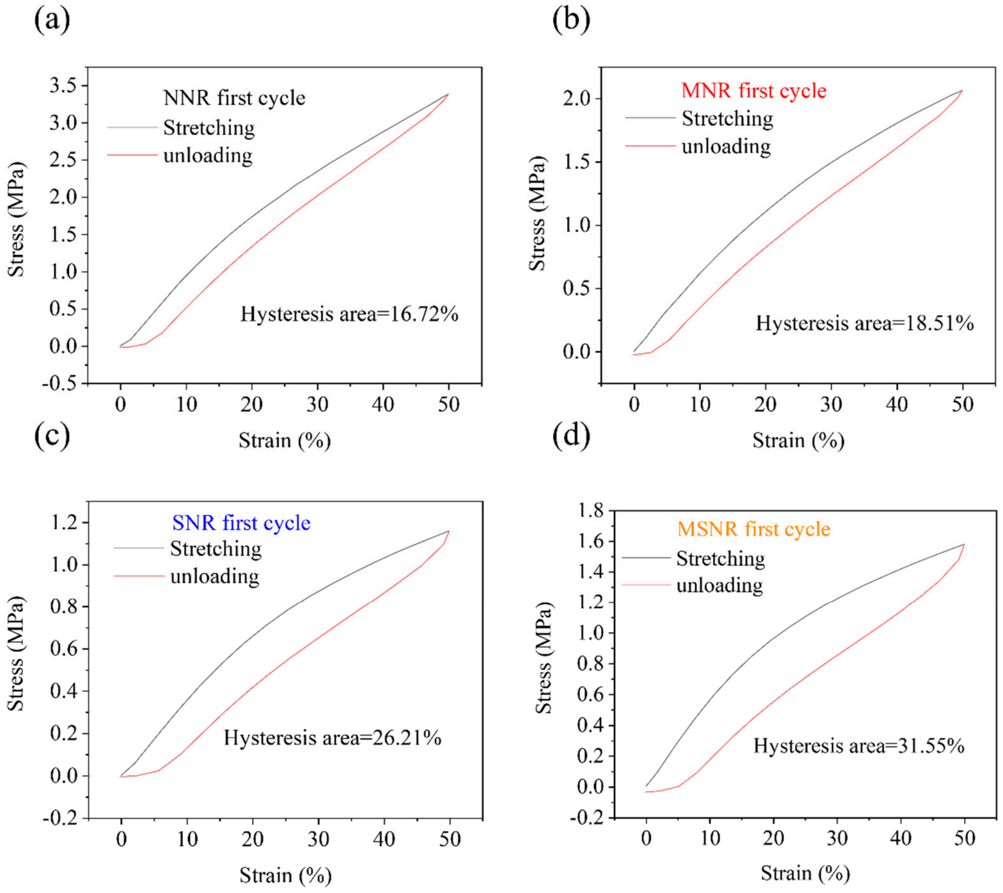

Figure S1. Stress–strain cyclic curve of NNR, MNR, SNR and MSNR.

Table S2. Comparison of mechanical properties.

Sample	Tensile strength (MPa)	Elongation at break (%)	Young’s modulus (MPa)
NNR	10.59	348.19	32.87
MNR	18.455	824.86	44.69
SNR	19.91	964.86	48.46
MSNR	24.2926	1218.2	50.14

Table S3. DMA performance comparison.

Sample	Storage modulus (MPa)	Loss modulus (MPa)	Tan δ
NNR	4639.11	270.52	0.1495
MNR	3519.15	296.01	0.1312
SNR	4727.26	311.84	0.1272
MSNR	5497.66	344.22	0.1183

Table S4. The formula of the composites.

Materials	Content
NR	4g
MWCNTs	0.2g
THF	500ML

SiO_2	0.12g
ZnO	0.2g
SA	0.08g
NS	0.06g
4010NA	0.08g

S2.1 MSD

In composites, if MWCNTs and SiO_2 are uniformly dispersed in the natural rubber latex matrix, this usually results in lower MSD values because a better dispersion implies a stronger interaction between the filler and the matrix. This in turn inhibits excessive aggregation and movement of the filler particles.

S2.2 FFV

When MWCNTs and SiO_2 are added to a natural rubber latex matrix, if both are well dispersed, they will fill the voids in the matrix, reducing the free volume of the composite. A lower free volume means tighter alignment of the molecules, which increases the mechanical strength, modulus and durability of the composite. Higher FFVs (greater void volume fraction): This usually means that there are more voids or pores within the material, which facilitates the flow and penetration of gas molecules. As a result, a higher FFV usually leads to a higher permeability of the material. Lower FFVs (smaller void volume fraction): A lower FFV may restrict the free flow of gas molecules, resulting in a lower permeability. For dense materials, it is difficult for gas molecules to pass through internal pores or voids, thus reducing permeability. Higher FFVs: An increase in free volume usually means that there are more voids or defects in the material, which can lead to a decrease in the density of the material. The presence of voids tends to make the material softer or easier to deform, which affects its mechanical strength. Lower FFVs: The material has a denser structure with stronger intermolecular interaction forces, which usually results in a higher strength and stiffness. A lower FFV has a beneficial effect on the compressive strength, tensile strength and toughness.

S2.3 Binding energy

For natural rubber latex/filler composites, a high binding energy typically means a strong chemical or physical interaction between the MWCNTs and SiO_2 and the natural rubber latex molecular chains. This strong interaction helps to reduce agglomeration or deposition of the filler, thereby improving its dispersion in the matrix. In molecular dynamics simulations, the binding energy is usually a combined effect of van der Waals and electrostatic forces that interact and together determine the stability and mechanical properties of the material.

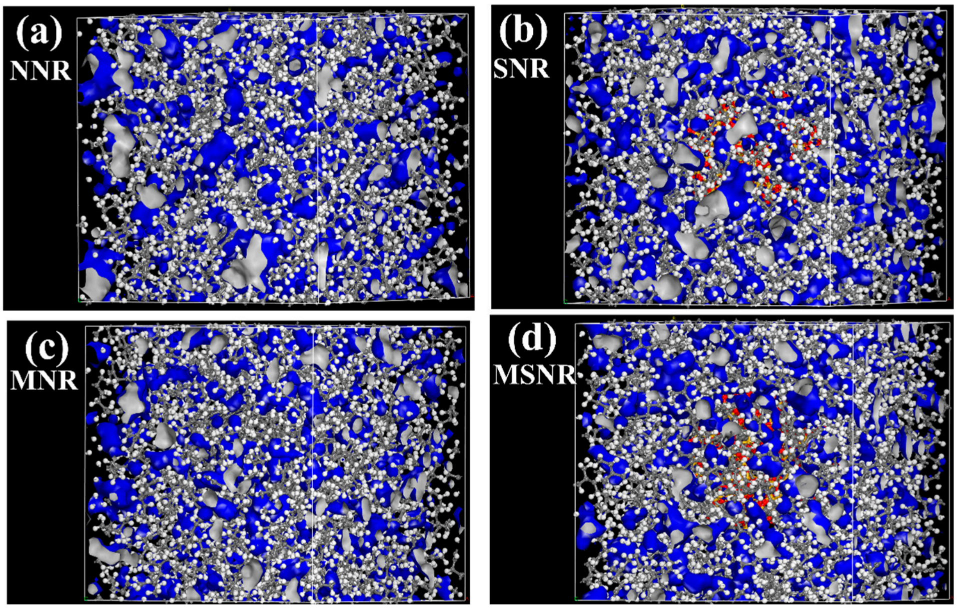

Figure S2. Free volume model of NNR, MNR, SNR and MSNR.

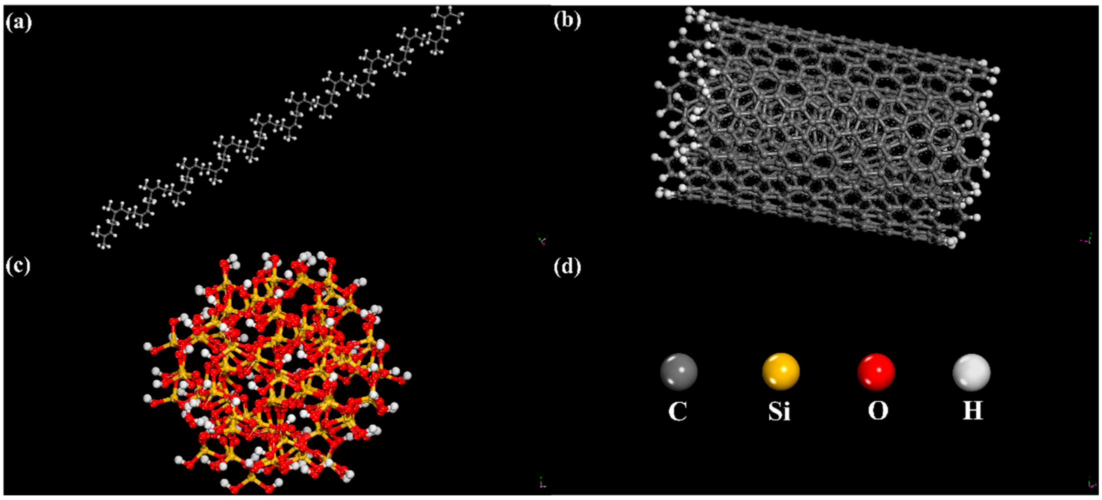

Figure S3. Molecular dynamics model: (a) NR; (b) MWNCT; (c) SiO2 and (d) different atoms.
